# Evaluation of the Prevalence of Refractive Defects and Ocular Function in a Group of 1518 Children Aged 8 Years in Northwestern Poland—A Retrospective Study

**DOI:** 10.3390/jcm12082880

**Published:** 2023-04-14

**Authors:** Modrzejewska Monika, Magdalena Durajczyk

**Affiliations:** Department of Ophthalmology, Pomeranian Medical University in Szczecin in Poland, Al. Powstańców Wlkp. 72, 70-111 Szczecin, Poland

**Keywords:** refractive errors, myopia, pseudomyopia, accommodative spasm, 8-year-old elementary school children

## Abstract

Purpose: To determine the prevalence of refractive errors in a group of 8-year-old school children in northwestern Poland. Material and Methods: In 2017–2019, refractive errors were examined in a group of 1518 Caucasian children aged 8 years old with cycloplegia. Refraction was obtained with a hand-held autorefractor (Retinomax 3). The refractive error reading was expressed as the spherical equivalent (SE) as myopia (≤−0.5 D), emmetropia (>−0.5 D to ≤+0.5 D), mild hyperopia (>+0.5 D to ≤+2.0 D) and hyperopia (>+2.0 D), and astigmatism (≤−0.75 DC) and anisometropia (≥1.00 D). Data analysis was performed using Statistica 13.5 software and included Pearson’s chi-squared and Mann–Whitney U tests. *p*-values of <0.05 were considered statistically significant. Results: Mild hyperopia was most common (37.6%), myopia was observed in 16.8% and astigmatism in 10.6% of participants. Pseudomyopia concerned up to 51.91% children. Girls were significantly more likely to have mild hyperopia (*p* = 0.0144) and were significantly more likely to wear glasses (*p* = 0.00093). Conclusions: Screening children for refractive errors after cycloplegia is key for detecting accommodative spasm and refractive errors. The largest group of children presented with mild hyperopia, which is a physiological feature of refraction in 8-year-old children, but myopia and astigmatism were the most common refractive errors.

## 1. Introduction

Refractive defects in children and adolescents are frequently analyzed by researchers due to their prevalence in the population and because they are a direct cause of negative socioeconomic consequences [1,2,3]. The WHO and the International Agency for the Prevention of Blindness have classified refractive defects as the second most common cause of blindness (after cataracts) and, in 1999, created an initiative called “Vision 2020: The Right to Sight”. Refractive defects are the cause of visual impairment in 88.4 million people worldwide [4]. The concept of good vision is related not only to normal visual acuity, but also relies on correct binocular vision, accommodation, oculomotor abilities and image processing in the visual cortex [5]. The high prevalence of visual impairments and the lack of widespread ophthalmic screening examinations to detect refractive defects in school-aged children have resulted in a certain group of children not achieving normal visual acuity as early as school age. The lack of correction of refractive disorders in this period of life leads to clinical symptoms, such as headaches, postural disorders and problems with concentration [6,7]. The lack of proper eye correction in refractive disorders can lead to strabismus and disorders in accommodative tension [8]. The authors of this study present the results of refractive disorders in a group of 8-year-old children, which were obtained during screenings carried out in elementary schools. We also emphasize the significant prevalence of refractive defects already in this age group, which indicates the need for ophthalmic screening, especially among the youngest school-aged children since most cases of myopia develop after children start elementary school [9]. The purpose of this study was to evaluate the prevalence of refractive defects and the function of the visual organ, including eye alignment and accommodation, as well as binocular vision, in a population of 1518 Polish 8-year-old children attending elementary school.

## 2. Definitions

The World Health Organization (WHO) and the International Classification of Diseases (ICD-10) defines myopia as a refractive error in which light rays running parallel to the optical axis are focused in front of the retina when accommodation is in a relaxed state [10]. This is because the refractive state of the eye is determined by the axial length of the eyeball in relation to the refractive power of the eye’s optical system. Epidemiological studies have shown that the prevalence of myopia in children varies around the world depending on geographic location, but also on the age of these children—from 1.9% in Northern Ireland to as much as 84% in Taiwan [11]. The results show that the prevalence of this refractive defect is steadily increasing [11]. According to the IMI (Interventions for Controlling Myopia Onset and Progression Report), the current threshold value for myopia is a spherical equivalent refractive defect of ≤−0.50 diopters and is stratified into low myopia (−0.5 and >−6.00 D) and high myopia (≤−6.00 D) [12]. This criterion takes into account refractive testing after accommodative paralysis, which is particularly important in children since a high gradient of accommodative tension is observed in this age group, the contraction of which leads to so-called pseudocorneal myopia. Hence, the refractive assessment of the eye without accommodative paralysis, which is commonly presented in the literature, can be unreliable due to the different negative values obtained most often in the range of −0.5 to −2 spherical diopters [13,14]. Accommodative spasm and pseudomyopia refer to excessive contraction of the ciliary muscle, which clinically manifests as blurred vision, distorted images, photophobia and eyeball pain. Symptoms are variable and can occur either monocularly or binocularly and continuously or episodically [15]. The condition is called pseudohyperopia and, unlike high myopia, is associated neither with an increased axial length of the eyeball nor excessive curvature of the cornea or lens [16]. Hyperopia is a refractive error in which light rays running parallel to the optical axis are focused behind the retina when accommodation is in a relaxed state [17]. Hyperopia is a common refractive error occurring in about 12% of young children depending on the population and the adopted definition [18,19]. Moderate to severe hyperopia (>3.00 D) adversely affects visual development and can be a precursor to accommodative esotropia, varifocality and mono- or bi-lateral visual impairment [20]. Astigmatism is a refractive error caused by optical aberration of the cornea and/or lens from their ideally spherical configurations [21]. Uncorrected astigmatism can lead to the progression of myopia and visual impairment [22,23,24] and can also cause visual developmental disorders in children, exacerbating blurred vision on the retina and headaches, which are observed in children younger than 15 [25,26]. The IMI pays particular attention to the refractive range in children from +0.75 D to >−0.50 D, called pre-myopia, in which the combination of the underlying refractive defect, age and other measurable risk factors for developing myopia carries a sufficient probability of developing myopia in the future and therefore merits preventive intervention [10]. For the aforementioned study, the Spherical Equivalent of Cylindrical Power (SNO), calculated as the sum of half the cylindrical power and spherical power expressed in spherical diopters (Dsph), was used as the criteria to assess refractive defects in children after total cycloplegia [27]. Based on the research of Morgan et al. [28], Helle K. Falkenberg et al. [29] and Flitcroft et al. [10], among others, the criteria for the diagnosis of refractive defects were adopted, as outlined in Table 1.

## 3. Materials and Methods

A total of 1518 eight-year-old second-grade students residing in Szczecin, a city in northwest Poland, were invited to participate in an eye examination to estimate the prevalence of refractive defects. These screenings were carried out from 2017 to 2019. The research project received a positive opinion from the Bioethics Committee of the Pomeranian Medical University in Szczecin (KB.006.25.2023). The research was conducted in accordance with the tenets of the Declaration of Helsinki after gaining consent from the legal guardian of the child undergoing screening.

The ophthalmologic examination included an assessment of the following ophthalmologic parameters: visual acuity at near and far distance (Snellen charts), two-fold examination of refractive defects using an autorefractometer (Retinomax3rd, SN 2202005 Tokyo, Japan 2012) before and after the use of cycloplegic drops (Tropicamide 1% at 1 drop 3 times every 5 min according to the standards of accepted refractive tests, tropicamide can be considered a viable substitute for cyclopentolate) [27], evaluation of the anterior segment of the eye in a hand-held slit lamp, evaluation of the fundus (direct ophthalmoscopy) by means of an ophthalmoscope speculum and indirect examination of the fundus (Fison speculum), evaluation of accommodation (examination of the near point of each eye (three-fold repetitions), three-fold examination of convergent accommodation, evaluation of eye alignment and muscular balance (unilateral and alternating cover-test), examination of ocular mobility in 6 directions of gaze, examination in the direction of exophoria by means of the Maddox test for a distance of 5 m and the Maddox test for a distance of 30 cm. A Titmus Test was performed to assess spatial vision. In addition to the ophthalmic examination, the child’s caregivers filled out a questionnaire with questions about ocular complaints, eyeglass wear, history of ocular surgery (strabismus, glaucoma, cataracts) and the presence of myopia and other ophthalmic diseases in the family. This article focuses on evaluating the results of refractive errors.

Statistical analysis of the data was performed using Statistica 13.5 and included Pearson’s chi-squared and Mann–Whitney U tests. Values of *p* < 0.05 were considered statistically significant.

## 4. Results

**Figure 1:**
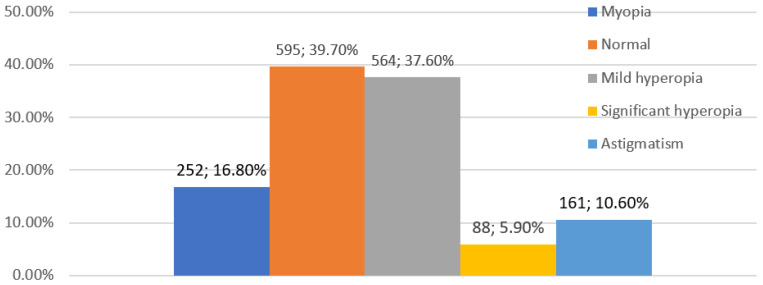
Prevalence of refractive errors after cycloplegia in 8-year-old children in the study group.

**Figure 2:**
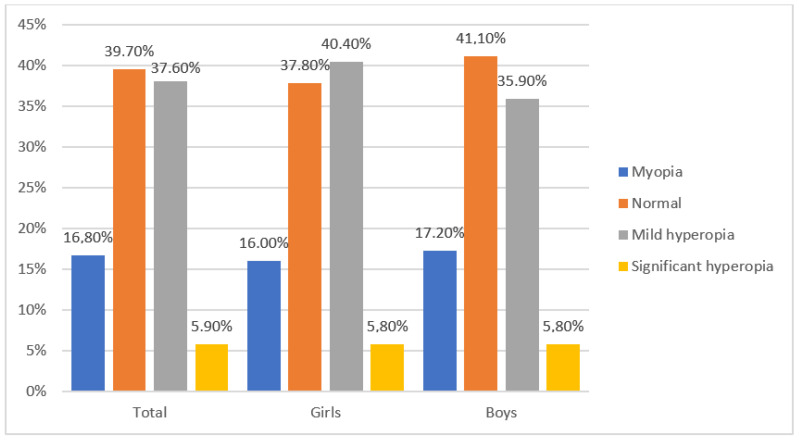
Prevalence of refractive errors after cycloplegia in the study group according to gender.

The study included 1518 elementary school second-grade children (*n* = 3036 eyes) of whom 727 (48%) were girls and 791 (52%) were boys. The average age of children entering the study was 8.2 years (SD = 0.6). All the children surveyed were Caucasian from urban and suburban areas. Following the aforementioned criteria for classifying refractive defects in children (Table 1), the following results were obtained for the prevalence of refractive errors in the study group: mild hyperopia—564 children (37.6%), myopia—252 children (16.8%), astigmatism—161 children (10.6%) and significant hyperopia—88 children (5.9%). Emmetropia occurred among 595 children (39.7%) (Figure 1). Of those examined, 1407 (91.4%) showed full visual acuity (V = 1.0, according to Snellen), 1305 (85.9%) did not wear glasses, 83 (5.4%) had full visual acuity when wearing their glasses, 28 children (1.9%) showed abnormal vision when wearing their glasses (the visual acuity was worse than 1.0 in at least one eye) and 102 children (6.7%) requiring treatment had never worn glasses. A group of 130 children (8.6%) required treatment for abnormal refractive defect correction, abnormal vision or other ocular diseases. Among the refractive defects analyzed, mild hyperopia was more often observed in girls (Figure 2). Of the 1518 children, 111 (7.35%) wore glasses, including 70 girls (9.6%) and 41 boys (5.1%). A statistically significant difference (*p* = 0.00093) was observed between gender and wearing glasses. Anisometropia was present in 60 patients (3.98%).

**Figure 3:**
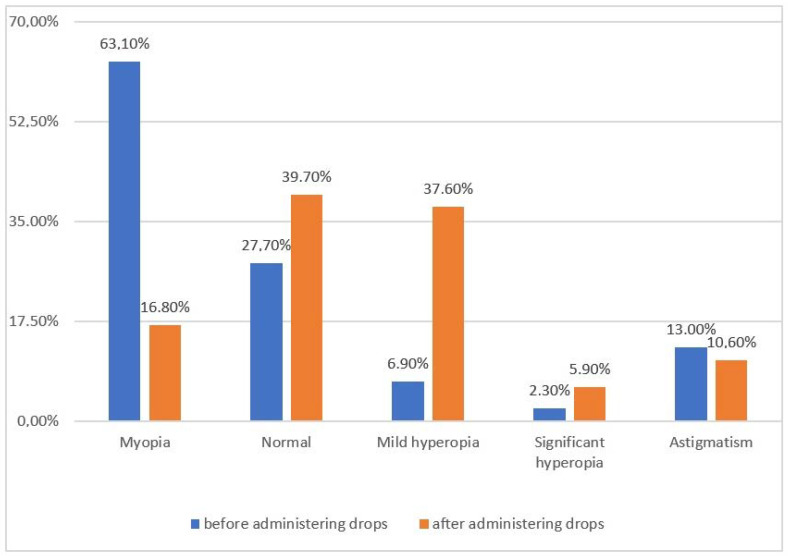
Difference in the magnitude of refractive errors before and after administration of accommodative paralysis drops.

Changes in the eye’s refraction, reported as differences in the values before and after the administration of cycloplegic drops, are included in Figure 3. In our study, 68.71% of the children met the myopia criterion before cycloplegia, whereas 16.8% met it after cycloplegia. 

This result indicates that apparent myopia affected 51.91% of the children. The mean spherical equivalent before the administration of the cycloplegic drops was −1.78 D, whereas after cycloplegia, it was −0.8 D.

## 5. Discussion

In our study, because we wanted to achieve a rapid and effective cycloplegic effect with minimal drug side effects and a rapid return of accommodation to baseline, we decided to use 1% Tropicamide administered three times at an interval of 5 min. One drop was administered into each eye. This method was suitable for use among learning children as its effect lasts for about 6 h [32]. 

The prevalence of refractive changes varies by race, age, gender and demographic region [33]. For the purpose of this article, in order to compare the prevalence of refractive errors in children in Europe and the world, the authors of this manuscript analyzed PUBMED meta-analyses data on the prevalence of refractive errors in children <18 years of age from selected countries from European and global populations from 2000 to 2018. The data is presented in Table 2 and Table 3.

In our screening study of 8-year-old elementary school second-grade children, emmetropia was diagnosed among 39.7%, a result similar to that obtained by Polish researchers in a screening study of children aged 6 to 15 years of age in 2002, who, after examining a group of 1002 students, diagnosed normopia in 34% of the study group [29]. The most common refractive error diagnosed in our study was mild hyperopia, followed by myopia, astigmatism and significant hyperopia. Similar results were obtained in the aforementioned study on Polish children conducted in 2002 [29] (Table 2). Similar results were obtained by researchers studying refractive errors in children in Nepal, India, China and South Africa in 2005 [30]. The size of the study population in these countries was 38,811 children aged 5 to 15 years. The percentage distribution of refractive errors in this study compared to our data was as follows: myopia, < 20% [30] and 16.8% in our study; emmetropia, 20–47% [30] and 39.7% in our study; and hyperopia, 15–73% [30] and 43.5% in our study (Table 3). The authors found that mild hyperopia was the most commonly diagnosed refractive value in early childhood, whereas myopia predominated in 15-year-old children. Emmetropia is classically defined as a condition between myopia and hyperopia in which parallel running light rays refract and focus on the retina with relaxed accommodation. There are many mechanisms of emmetropization. In humans, the process appears to be subject to feedback and involves coordinated changes in the corneal power and axial length of the eyeball [34]. Decreases in lens power have also been documented [35]. Next, corneal power undergoes stabilization. Subsequently, axial elongation of the eyeball occurs, combined with a decrease in lens power and an increase in anterior chamber depth, which may at least partially compensate for the myopic shift that is normally associated with axial elongation of the eyeball [30]. Cycloplegic findings suggest that the mean spherical equivalent value of refraction in childhood, as well as in young adults, is hyperopia, not emmetropia [36]. In other populations, such as China, where the prevalence of myopia is high, the average cycloplegic spherical equivalent value shifts decisively toward myopia by the end of childhood [37]. This is also related to, among other things, the fact that children in Hong Kong as young as 2 years old and in Singapore as young as 3 years old actively participate in supplementary educational activities before starting formal schooling [12].

In Europe, results similar to ours were published by Norwegian researchers in 2019 [38]. This was a retrospective study of 782 children aged from 7 to 15 years who were diagnosed with mild hyperopia (51%), emmetropia (32%) and myopia (17%). A significant limitation of this study is the failure to perform cyclopegia when examining refraction. Refraction, especially in children and young adults, is usually the result of accommodative contraction and possible myopic shift. Accommodative contraction occurs in the form of latent myopia (or otherwise pseudomyopia) or latent hyperopia. Therefore, refractive testing with cycloplegic drugs effectively reduces the fluctuation of accommodation, resulting in relaxation of the ciliary muscle [39]. In the study currently presented by the authors, the significant effect of cycloplegia on the diagnosis of the final refractive defect was demonstrated. Before accommodative paralysis, as many as 68.71% of children met the criteria for myopia, whereas after cycloplegia, this number decreased to 16.8%. It follows that, according to the study, as many as 51.9% of the children we examined had over-minus estimation when examined with an autorefractometer without cycloplegia. The results of the study confirmed the necessity of using cycloplegia in children to effectively reduce refractive pseudomyopia. The term “pseudomyopia” refers to a phenomenon that especially occurs in young children, denoting a refractive defect in the form of myopia that disappears after cycloplegia because it is still reversible [40]. Pseudomyopia is the result of an increase in the refractive power of the eye due to excessive stimulation of the accommodation mechanism [41,42]. It arises as a result of continuous overstimulation of the ciliary muscle, which does not relax completely when focusing the eye on an object at infinity [41]. The difference in the refractive power in eyes after cycloplegia and without cycloplegia is one of the diagnostic signs of pseudomyopia. In pseudomyopia, the refraction of the eyes in the absence of cycloplegia is more negative than after the administration of cycloplegic drops [41,43,44]. In our results, the absolute difference of the mean spherical equivalents before and after cycloplegia was 0.98 D, which is still within the criteria of reversible apparent myopia (<1.0 D) [45]. Recognizing pseudomyopia in children is extremely important because it is amenable to therapy. Furthermore, preventive measures can halt or slow the development of myopia. The results of a study published in 2019 showed that the administration of 0.05%, 0.025% and 0.01% atropine can prevent the progression of myopia [46], and most significantly, inhibited the development of refractive errors and an increase in axial eye length at a one-year of follow-up. In addition, other non-pharmacological approaches, such as the use of multifocal contact lenses [47] and orthocorrection [48], facilitate the prevention of myopia progression. In this study, a statistically significant difference was observed between gender and the wearing of glasses, which is also confirmed in the literature and is attributed to a greater emphasis on education and more frequent close work (reading, drawing and writing) in girls compared to boys [49]. Our study showed that as many as 102 children (6.7%) requiring treatment had never worn glasses. In the available literature, the authors point out that children’s reluctance to wear glasses is influenced by the fear of being bullied by those around them [50], the fear of compromising their appearance [51] and reduced attractiveness [52].

**Table 2:** Prevalence of refractive errors among children in Europe, according to the literature [38,53,54,55,56].

Author	Country and Year	Age Range in Years	Average Age	Size of Study Population	Cycloplegia	Refractive Error and Its Prevalence in Population
Popović-Beganović [53]	Bosnia and Herzegovina 2017	7–16		997	YES	Myopia 20.4% Hyperopia 3.3% Astigmatism 18.1%
Harrington SC [54]	Ireland 2016–2018	Two groups 6–7 12–13		1626	YES	Age group 6–7 years Myopia 3.3% Hyperopia 25% Astigmatism 19.2% Age group 12–13 years Myopia 19.9% Hyperopia 8.9% Astigmatism 15.9%
O’DomoghueL [55]	Northern Ireland 2006–2008	Two groups 6–7 12–13		392 (age group 6–7 years) 661 (age group 12–13 years)	YES	Age group 6–7 years Myopia 2.8% Hyperopia 26% Age group 12–13 years Myopia 17.7% Hyperopia 14.7%
Falkenberg [38]	Norway 2003–2013	7–15		1126	NO	Emmetropia 32% Hyperopia 51% Myopia 17%
Szaflik et al. [29]	Poland 2002–2003	6–16	10	10,021	YES	Emmetropia 33.77% Hyperopia 19.91% Myopia 16.94% Astigmatism 5.57% Complex vision errors 24.05%
Villarreal MG [56]	Sweden 2000	12–13		1045	YES	Myopia 39% Hyperopia 8.4% Astigmatism 5.2%

**Table 3:** Prevalence of refractive errors in children worldwide, according to the literature [2,30,57,58,59,60,61,62,63,64,65,66].

Author	Country and Year	Age Range in Years	Average Age	Size of Study Population	Cycloplegia	Refractive Error and Its Prevalence in Population
Morgan [30]	Nepal, India, Chile, South Africa 2005	5–15		38,811	YES	Myopia < 20% Emmetropia 20–47.2% Hyperopia 15–73%
RobaeiD [57]	Australia 2003–2004	5.5–8.4	6.7	1740	YES	Myopia 1.6% Hyperopia 13.2% Astigmatism 7.6% Normovision 80%
Ojaimi [58]	Australia 2005	6–7		1724	YES	Emmetropia 7.57% Hyperopia 91% Myopia 1.43%
Lira [59]	Brazil 2012	6–17		778	YES	Emmetropia 15.9% Hyperopia 74.6% Myopia 9.6%
MaulE [2]	Chile 2000	5–15		6998	YES	Myopia 7.3% Hyperopia 19.3% Astigmatism 27%
Li H, Li S, Liu L [60]	China 2014	12.9–17.6	14.7	1839	YES	Hyperopia 7.5% Myopia 82.7% Emmetropia 9.8%
Ma Y [61]	China 2016	3–10		8398	YES	Hyperopia 17.8% Myopia 20.1%
DandonaR [62]	India 2002	<15		11 786	YES	Hyperopia 62.62% Myopia 3.19%
GursoyH [63]	Iran 2011	7–8		709	YES	Myopia 22.6% Hyperopia 10.6% Astigmatism 11.0%
HashemiH [64]	Iran 2013	7		4072	YES	Myopia 3.04% Hyperopia 6.20% Astigmatism 17.43%
Yingyong [65]	Thailand 2008–2009	6–12		1100	YES	Myopia 11.1% Hyperopia 1.4% Astigmatism 0.3%
CacaI [66]	Turkey	6–14	10.56 ± 3.59	21.062	YES	Myopia 3.2% Hyperopia 5.9% Astigmatism 14.3%

## 6. Conclusions

In the studied group of Polish 8-year-old second-grade children, the most frequently detected refractive error after the application of cycloplegia was mild hyperopia, which is a physiological feature of refraction in 8-year-old children (37.6%). Statistically significant differences were observed between gender and the wearing of glasses, with a statistical advantage for the female gender (*p* = 0.00093).

Our results emphasize the importance of conducting regular vision screenings in children. It seems that the high percentage of myopic disorders associated with pseudomyopia, confirmed by the results of the current study, are likely related to digitization and an already excessive workload on near vision.

## Figures and Tables

**Table 1:** Criteria for refractive defects, according to International Myopia Institute, Morgan et al. [30], Helle K. Falkenberg et al. [29], D.I. Flitcroft et al. and Hu Y., Zhao F. [10,31].

Refraction Parameter	Spherical Equivalent Criteria (D)
Emmetropia	−0.5 to ≤+0.5
Pre-myopia	−0.50–+0.75
Myopia	≤−0.50
Low myopia	−0.50–6
High myopia	≤−6
Refractive myopia	a myopic refractive state that can be attributed to changes in the structure or location of the image-forming structures of the eye; that is, the cornea and/or lens
Axial myopia	a myopic refractive state that can be attributed to excessive axial elongation
Secondary Myopia	a myopic refractive state for which a single, specific cause (drug, corneal disease or systemic clinical syndrome) can be identified that is not a recognized population risk factor for the development of myopia
Pseudomyopia	instrument myopia, night myopia or accommodative spasm
Mild hyperopia	≥+0.50–≤+2.0
Significant hyperopia	≥+2.00
Anisometropia	≥1.00 (difference between eyes)
Astigmatism	≤−0.75 (cylinder)

## Data Availability

The data used to support the findings of this study are available from the corresponding author upon request.
